# Relationship between Peak Inspiratory Flow and Patient and Disease Characteristics in Individuals with COPD—A Systematic Scoping Review

**DOI:** 10.3390/biomedicines10020458

**Published:** 2022-02-16

**Authors:** Marika T. Leving, Janwillem Kocks, Sinthia Bosnic-Anticevich, Richard Dekhuijzen, Omar S. Usmani

**Affiliations:** 1 General Practitioners Research Institute, 9713 GH Groningen, The Netherlands; janwillem@gpri.nl; 2 GRIAC Research Institute, University Medical Center Groningen, University of Groningen, 9700 RB Groningen, The Netherlands; 3 Observational and Pragmatic Research Institute, Singapore 573969, Singapore; 4 Woolcock Institute of Medical Research, Sydney, NSW 2037, Australia; sinthia.bosnic-anticevich@sydney.edu.au; 5 Sydney Pharmacy School, Faculty of Medicine and Health, University of Sydney, Sydney, NSW 2006, Australia; 6 Sydney Local Health District, Sydney, NSW 2050, Australia; 7 Radboud University Medical Center, 6525 GA Nijmegen, The Netherlands; richard.dekhuijzen@radboudumc.nl; 8 National Heart and Lung Institute (NHLI), Imperial College London, London SW3 6LY, UK; o.usmani@imperial.ac.uk; 9 Royal Brompton Hospital, London SW3 6NP, UK

**Keywords:** peak inspiratory flow, COPD, suboptimal, determinants, patient characteristics, systematic scoping review, inhalation therapy

## Abstract

Optimal delivery of medication via dry powder inhalers, the most commonly prescribed inhaler type, is dependent on a patient achieving a minimum level of inspiratory flow during inhalation. However, measurement of peak inspiratory flow (PIF) against the simulated resistance of a dry powder inhaler is not frequently performed in clinical practice due to time or equipment limitations. Therefore, defining which patient characteristics are associated with lower PIF is critically important to help clinicians optimize their inhaler choice through a more personalized approach to prescribing. The objective of this scoping review was to systematically evaluate patient and disease characteristics determining PIF in patients with chronic obstructive pulmonary disease (COPD). Medline, Cochrane and Embase databases were systematically searched for relevant studies on PIF in patients with COPD published in English between January 2000 and May 2021. The quality of evidence was assessed using a modified Grading of Recommendations Assessment, Development and Evaluation checklist. Of 3382 citations retrieved, 35 publications were included in the review (nine scored as high quality, 13 as moderate, nine as low, and four as very low). Factors correlating with PIF in >70% of papers included both patient characteristics (lower PIF correlated with increased age, female gender, shorter height, decreased handgrip and inspiratory muscle strength, and certain comorbidities) and disease characteristics (lower PIF correlated with markers of lung hyperinflation, lower peak expiratory flow [PEF] and increased disease severity). Other factors correlating with adequate/optimal or improved PIF included education/counseling and exercise/inspiratory muscle training; impaired physical function and errors in inhalation technique/non-adherence were associated with low/suboptimal PIF. In conclusion, clinicians should measure PIF against the simulated resistance of a particular device wherever possible. However, as this often cannot be done due to lack of resources or time, the patient and disease characteristics that influence PIF, as identified in this review, can help clinicians to choose the most appropriate inhaler type for their patients.

## 1. Introduction

Chronic obstructive pulmonary disease (COPD) is a chronic lung condition commonly caused by prolonged exposure to tobacco smoke, biomass fuels and/or air pollution and characterized by inflammation and peripheral airway narrowing, which leads to airflow limitation [1]. This is compounded by parenchymal destruction (emphysema) and loss of small airway function in the lung [1]. COPD is associated with persistent respiratory symptoms such as breathlessness, cough, and sputum production [1], which fluctuate throughout the day [2].

Inhaled therapy is the cornerstone of treatment for COPD, with four main classes of delivery systems: pressurized metered-dose inhalers (pMDIs), dry powder inhalers (DPIs), soft mist inhalers and nebulizers [3]. Of these, pMDIs and DPIs are the two most widely prescribed inhaler types [4], and the current trend towards more eco-conscious prescribing may further increase uptake of DPIs, which have a lower carbon footprint than pMDIs [5,6]. Optimal delivery of inhaled therapy, among other factors, depends on the ability of patients to generate sufficient peak inspiratory flow (PIF) [7]. This is especially important for DPIs, as this class of delivery system has an internal resistance, which needs to be overcome for particles to disaggregate and reach the lungs [7,8]. Insufficient inspiratory flow can lead to poor disaggregation of the drug from its carrier particles and/or deagglomeration (for drug-only formulations), resulting in greater deposition in the oropharynx rather than the target site in the lungs [7,9]. Depending on the magnitude of the inhalation flow, drug deposition in the lungs with DPIs has been reported to be as low as 15% [9].

Suboptimal PIF and poor clinical outcomes in patients have been reported in some studies [8,10,11]. For example, in hospitalized patients with COPD, PIF ≤60 L/min at discharge was associated with a shorter time to all-cause and COPD-related hospital readmission compared with PIF >60 L/min [11]. In addition, reduced PIF has been associated with a worse burden of symptoms related to COPD [8,11]. However, drawing robust conclusions about the effect of PIF alone on disease outcomes is complicated by the complex interplay between other factors such as inhalation technique and adherence.

It is important to assess PIF against the simulated resistance of a DPI to ensure that a patient is capable of achieving optimal drug deposition; however, this is not commonly carried out in clinical practice [12,13]. PIF can be measured using several methods, such as flow volume measurements as part of routine spirometry; however, this type of measurement is conducted at zero resistance and may be misleading [12,14]. Devices such as the In-Check DIAL provide inexpensive and easy-to-use alternatives with the option of simulating the resistance of various inhalers and thus measuring PIF in a more useful clinical context [12,14]. However, as PIF determined from flow-volume measurements may have only weak or moderate correlations with PIF measured with the In-Check DIAL, it is important to check PIF against a particular DPI resistance before prescribing [12,14]. Adoption of standardized recommendations for PIF measurement can help to ensure accurate and reliable PIF values, both in clinical trials and in daily clinical practice. [15].

In those frequent situations where it is not possible to measure PIF against the simulated resistance of a particular inhaler, it is important for clinicians to keep in mind patient and disease characteristics that might predict a patient’s inspiratory flow. The available literature on this topic is limited, but there is some evidence that patient characteristics such as age, gender, height and patient inspiratory effort, alongside disease characteristics such as inspiratory capacity (IC), disease severity and acute exacerbations of COPD, may influence PIF [16,17,18,19,20]. Furthermore, PIF may be reduced in patients with generalized muscle weakness, for example, in those with reduced handgrip strength [21], as well as in patients with prolonged hyperinflation, regardless of peripheral muscle function [22]. However, to our knowledge, no study has systematically reviewed and synthesized the available literature on the determinants of PIF.

The aim of this systematic scoping review was to identify the key determinants of PIF, in terms of both patient factors and disease characteristics, in patients with COPD, to help clinicians in their choice of inhaler. This is critically important if we are to advance clinical practice and optimize the use of inhalers in the real-world setting through a more personalized approach to inhaler prescribing and use.

## 2. Materials and Methods

### 2.1. Population of Interest

Our focus was patients with COPD, although some of the identified articles included patients with asthma or other lung-related diseases. In these instances, we included data that focused only on patients with COPD and disregarded data on patients with asthma and asthma–COPD overlap or data sets where the populations of patients with COPD and asthma were combined. 

### 2.2. Information Source

The Medline, Cochrane and Embase databases were searched systematically for relevant studies published in the English language between January 2000 and May 2021. The search terms were compiled from words related to COPD and PIF (Supplementary Table S1). Duplicate articles were excluded during initial screening of the compiled lists. Articles were screened by two separate assessors to determine their relevance to the research question; any discrepancies between the first two assessors were resolved by a third reviewer. A manual search of references cited in selected review articles was also performed. 

### 2.3. Eligibility Criteria

Studies were included in the data analysis if they met the following criteria: (1) published in English; (2) included mention of PIF in the title or abstract; (3) mentioned factors associated with PIF (either patient- or disease-related); and (4) published after 1 January 2000. Published articles were excluded if they: (1) were congress articles or abstracts, reviews, letters to the editor or responses; or (2) did not mention factors associated with PIF. Articles were excluded if standalone data were not available on patients with COPD.

Once articles had been identified according to the inclusion and exclusion criteria, they were screened during the full data charting stage.

### 2.4. Data Charting and Quality Assessment

One assessor extracted the following information from each included study: Title, year of publication, patient-related characteristics associated with PIF, disease-related characteristics associated with PIF, and other factors associated with PIF; then a second assessor checked and verified the extracted data.

Although not typical for a systematic scoping review [23], we also conducted a quality assessment of data to indicate the strength of the evidence compiled. The quality of evidence from each study was independently determined by two separate assessors using a 6-item checklist relating to data and methods. A tailored approach was adopted to assess the quality of the selected articles, incorporating principles from the established assessment framework, namely Grading of Recommendations Assessment, Development and Evaluation (GRADE) [24], covering study type, study design/execution, population relevance, imprecision, magnitude of effect and the impact of confounding factors. Further details on the methodology, including modifications to GRADE, are provided in the Supplementary Methods. Using the adapted GRADE criteria, individual items were assigned a positive score if they were likely to improve the quality of the evidence, and a negative score if they were likely to decrease the quality of the evidence. The scores were then used to derive the final quality assessment score and grade; the evidence was reported as high, moderate, low or very low quality, as defined in Supplementary Table S2. 

### 2.5. Statistical Analysis

Statistical analysis was not conducted in this systematic scoping review. Due to heterogeneity in the study design of the included publications, a meta-analysis was not possible. 

## 3. Results

### 3.1. Selection of Papers

**Figure 1:**
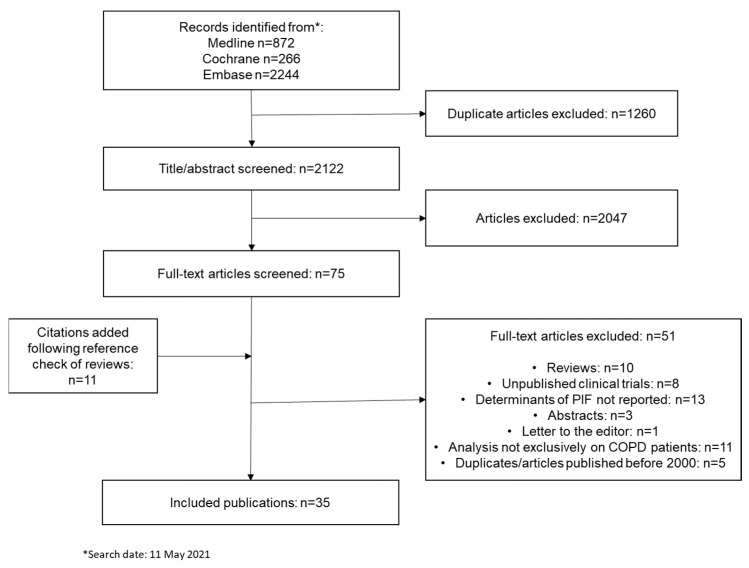
PRISMA flowchart illustrating the selection process of articles.

A total of 3382 citations were retrieved from the Medline, Embase and Cochrane databases, of which 2122 unique articles were identified. Screening of the title and/or abstracts of these articles led to the inclusion of 75 full-text articles. A further 11 articles identified from reference lists of review papers were also screened. In total, 35 publications were considered eligible and included in the review (Figure 1).

### 3.2. Quality Assessment of Papers

Of the 35 publications assessed, nine were scored as high quality (very confident in the effect estimate), 13 as moderate (moderately confident), nine as low (limited confidence), and four as very low (very limited confidence) (Supplementary Tables S2 and S3). The majority of articles (32/35) explored the relationship between PIF and patient/disease characteristics as a key objective of the study and were assigned positive scores in terms of study design. Thirteen out of 35 included a population of patients that was considered sufficiently representative of the general COPD population, whereas the remaining articles included more specific subsets of patients (e.g., the elderly only or patients who had been hospitalized following an acute exacerbation of COPD), and thus received negative scores. The overall risk of imprecision arising due to, for example, small sample size, overlapping of wide confidence intervals and a lack of statistical analysis conducted to assess the correlation of PIF with patient/disease characteristics was relatively high across the studies, with 17/35 publications receiving a negative score for this item. The magnitude of the effect was scored positively for the majority of articles (24/35), suggesting that the effects observed in those articles were generally large and consistent. Potential confounding factors were identified for most articles (27/35), and these were generally not adequately discussed or controlled for, thus scoring negatively.

### 3.3. Definition and Measurement of PIF

In the studies included in this analysis, PIF refers to maximal inspiratory flow generated during a real or simulated inhaler maneuver. The cut-off for suboptimal/low and optimal PIF varied between studies (if reported). Most commonly, suboptimal PIF was defined as <60 L/min whereas optimal was >60 L/min; some studies had bespoke cut-offs tailored according to study objectives. The In-Check DIAL (including a modified device) was most commonly used to measure PIF (*n* = 17) [11,16,18,19,21,22,25,26,27,28,29,30,31,32,33,34,35], followed by a spirometer (including pneumotachographs and bespoke setups linked to software or fitted with a flowmeter; *n* = 12) [17,36,37,38,39,40,41,42,43,44,45,46], an inhalation profile recorder (*n* = 3) [47,48,49], and other devices such as a pressure transducer (*n* = 1) [50] and an In-Check Meter (*n* = 1; Supplementary Table S4) [51]. All studies were conducted in a clinical or laboratory setting under supervision.

### 3.4. Association of PIF with Patient or Disease Characteristics

Table 1 summarizes the key papers supporting or not supporting an association between PIF and various patient or disease characteristics. More details on data charting for all of the papers included in the systematic scoping review can be found in Supplementary Table S4.

**Key Quality Assessment Bar.** The overall breakdown of the quality assessment grades assigned to papers supporting/not supporting a given association are shown below.

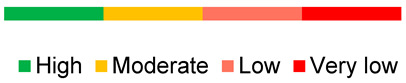



#### 3.4.1. Patient Characteristics

Of the 17 papers evaluating the association between age and PIF, 12 (71%) found an inverse correlation between age and PIF, such that increasing age was associated with decreasing PIF [11,17,21,22,25,28,30,32,34,35,44,47]; for the remaining five (29%), no association was found [18,19,29,36,46]. Chen et al. observed that patients aged >75 years had a significantly higher prevalence of suboptimal or insufficient PIF compared with younger patients when measured against DPIs with medium-high resistance; however, a significant correlation was not observed for DPIs with medium, medium-low, and low resistance [25]. Multivariate analysis by Jarvis et al. also demonstrated that the effect of age on PIF was independent of disease severity [30].

For gender, 14 papers evaluated the association with PIF. Of these, nine (64%) found a positive correlation between female gender and low PIF [17,18,19,22,26,32,35,36,47], and one (7%) noted a correlation between gender and PIF but did not state which gender was associated with lower PIF [16]; four (29%) did not find an association [11,21,29,34]. In one study of 303 patients with COPD, both gender and height were found to be independent predictors of suboptimal PIF; however, after further modeling of the findings, the effect on gender was no longer significant, suggesting that the impact of gender may be mediated by short stature [16]. For height, eight of the 11 papers (73%) evaluating an association with PIF found a positive correlation between shorter height and low PIF [16,17,18,19,22,28,32,44]; for the remaining three (27%), an association was not found [11,25,35].

Twelve papers evaluated the association between body weight, body mass index (BMI) or body composition, and PIF; of these, only two (17%) reported a correlation with low PIF (one with reduced body weight [17] and one with reduced fat-free mass, which is a marker of reduced muscle mass [37]). No association was reported in the remaining 10 papers (83%) [11,18,19,22,28,29,32,34,36,44]. 

Out of four papers evaluating the association between manual/muscle strength (handgrip strength [21] or maximal inspiratory mouth pressure, a measure of inspiratory muscle strength [26,27,28]) and PIF, all of them reported a positive correlation (100%).

Four papers evaluated the association between comorbidities and PIF; positive correlations were reported between low PIF and asthma [21], anemia [32], coronary artery disease [32], pneumonia [35], and ischemic heart disease [35]. However, Samarghandi et al. [21] did not report a correlation for coronary heart disease, hypertension, diabetes mellitus, or congestive heart failure; nor did Davidson et al. [32] for hypertension, depression, heart failure, or dementia/Alzheimer’s disease; nor did Sharma et al. [35] for congestive heart failure, hypertension, history of myocardial infarction, atrial fibrillation, diabetes, emphysema, asthma, chronic bronchitis, bronchiectasis, tuberculosis, depression, anxiety, osteoporosis or cancer. Loh et al. found no correlation between PIF and Charlson Comorbidity Index [11]. 

Of the five papers evaluating the smoking status, only one reported a correlation between current smoking and low PIF [35]; the other four papers did not report an association [21,29,34,36]. For ethnicity, no correlation was observed for any of the three papers evaluating the association between ethnicity and PIF [11,21,32].

#### 3.4.2. Disease Characteristics

Though limited in number, studies reported a consistent association between lower PIF and markers of lung hyperinflation, i.e., reduced IC (2/2 papers) [16,27], IC% predicted (3/4 papers) [11,16,22], vital capacity (VC) (1/1 paper) [27] and total lung capacity (TLC) (2/2 papers) [16,48]. Two thirds of studies (64%) reported a positive correlation between lower PIF and reduced forced expiratory volume in 1 second (FEV_1_) (9/14 papers) [16,17,19,25,32,34,36,44,48], though far fewer (19%) reported the same for FEV_1_% predicted (3/16 papers) [30,32,44]. Correlations were also observed between PIF and forced vital capacity (FVC) (70%; 7/10 papers) [16,25,28,32,34,36,44] and FVC% predicted (27%; 3/11 papers) [16,22,34]. Low PIF was correlated with lower values for a range of other parameters, though studies were few in number (*n* = 1–5): peak expiratory flow (PEF) [17,19,28,44,48], diffusing capacity of the lung for carbon monoxide (DL_CO_) [48], maximal expiratory pressure (MEP) [28] (although no correlation was reported in Terzano et al. [27]), forced inspiratory volume (FIV) [19], forced inspiratory vital capacity (FIVC) [19,44], maximum voluntary ventilation (MVV) [19], maximal forced inspiratory flow (FIF_max_) [16] and an increase in residual volume (RV)/TLC ratio [16]. 

For disease severity, of 11 papers evaluating an association with PIF, nine (82%) reported a correlation between increasing disease severity and low PIF [19,26,30,33,40,41,47,48,50]; two (18%) reported no association [18,36]. However, statistical analysis was not reported in the majority of these papers [18,26,33,40,41,47,50]. There was also heterogeneity in classification of disease severity in these studies: Global Initiative for Chronic Obstructive Lung Disease (GOLD) spirometry classification (GOLD stages I–IV) was used in three studies [18,47,48], GOLD grade A–D was used in two studies [19,36], and custom and/or study-specific definitions were used in the rest [26,30,33,40,41,50] (Supplementary Table S4). 

Out of three papers evaluating the association between exacerbations and PIF, one reported a correlation between exacerbation history and low PIF [34], but two reported no association [32,45]. Of note, in patients with ≥2 exacerbations in the previous year, Represas-Represas et al. reported a significant difference between the proportion of patients with adequate versus suboptimal PIF (25% vs. 47%, respectively); however, the difference for patients with adequate/suboptimal PIF was not significant in patients with no exacerbations in the previous year [34].

Seven papers evaluated the association between symptom burden (measured using COPD Assessment Test™ [CAT] or modified British Medical Research Council [mMRC] dyspnea scale scores) and PIF. Of these, only two noted a correlation between increased CAT scores and low PIF [11,19], whereas five reported no association with either CAT or mMRC scores [18,21,32,34,35].

#### 3.4.3. Other Factors

Training techniques were also found to correlate with increased PIF; of the five papers evaluating the association between enhanced educational training, instruction, verbal training or counseling with PIF, all found a positive correlation in that training improved PIF [31,33,49,50,51]. 

A positive correlation between exercise training or inspiratory muscle training and improved PIF was reported by Chen et al. [52] and Weiner et al. [26], respectively, but Tout et al. [39] did not observe an association between respiratory physiotherapy or inspiratory muscle exercise and PIF. Other factors reported to correlate negatively with PIF included inhalation technique errors, unconscious adherence and a lower activities of daily living (ADL) score, indicating worse functional status [32,34]. Factors for which no association with PIF were found included length of stay in hospital, inhaled corticosteroid use and left ventricular ejection fraction [11], primary education and feeling of satisfaction with the inhaler as determined by patient-completed questionnaire (Feeling of Satisfaction with Inhaler questionnaire) [34], pursed lip breathing [38], airway conductance [42] and arterial pH, partial pressure of oxygen and fraction of inhaled oxygen [29].

## 4. Discussion

To our knowledge, this is the first review of the available literature that has been carried out to identify the key determinants of PIF (patient and disease characteristics) in patients with COPD, which should help clinicians in selecting the most appropriate inhaler for their patients. Although we could not provide more detailed analysis at this time, we feel that our review represents the current knowledge and identifies what the focus of future studies should be. Therefore, it not only has clinical implications but also contains research recommendations that will guide future knowledge creation.

Patient factors that correlated with lower PIF in >70% of relevant papers in our review included increased age, female gender, shorter height, and decreased handgrip strength/mouth pressure (Table 1). Of the four studies that evaluated the relationship between the presence of comorbidities and lower PIF, three identified correlations with individual comorbidities, including asthma, anemia, pneumonia, coronary artery disease and ischemic heart disease (*n* = 1 for all); however, findings were heterogeneous, with other studies reporting no significant relationship between lower PIF and asthma (*n* = 1) and with various cardiovascular conditions (*n* = 3). Overall, no relationship was found between lower PIF and the majority of individual comorbidities examined in these studies [11,21,32,35]. Body weight/BMI/body composition, smoking status and ethnicity were only found to correlate with PIF in ≤20% of relevant papers.

In terms of disease characteristics, impaired lung function and increased disease severity correlated with lower PIF in a number of papers (Table 1). Markers of hyperinflation (reduced IC, IC% predicted, VC and TLC) were correlated with lower PIF in 75–100% of relevant papers, though these were few in number (*n* = 5). A relatively high proportion of studies (64–70%) reported a correlation between absolute FEV_1_ and FVC values and PIF, though a much lower proportion (19–27%) for the percent predicted values of these parameters. This may be due to the dependence of percent predicted values on age, gender and height [53], all of which are associated with PIF [16,17] and may therefore have reduced the chance for a correlation between percent predicted lung function parameters and PIF in our analysis. Lower PIF was also correlated with lower lung function values for DL_CO_, MEP, FIV, MVV and FIF_max_, as well as a higher RV/TLC ratio, though it should be noted that each was evaluated in a single study. Exacerbations correlated with lower PIF in one out of three relevant publications, where the study reported an association with lower PIF in patients with ≥2 exacerbations in the previous year, but no association in patients with no exacerbations in the previous year [34], suggesting a relationship may exist between exacerbations and lower PIF depending on the exacerbation threshold. Factors correlating with adequate/optimal or improved PIF in our analysis included education/counseling and exercise training/inspiratory muscle training, whereas worse physical function and errors in inhalation technique/non-adherence were associated with low/suboptimal PIF rate (Table 1). However, for many of these factors, the number of papers evaluating an association was very low and the study quality was heterogeneous.

Except for the associations observed for PEF (*n* = 5), education/counseling (*n* = 5), handgrip strength/inspiratory muscle strength (*n* = 4), IC (*n* = 2), FIVC (*n* = 2) and TLC (*n* = 2), no other single factor supported by at least 2 studies had a 100% consistent association with PIF. This may be due in part to the complexity of the relationship between patient/disease characteristics and PIF, and in part due to the heterogeneous nature of the data, with variable patient populations and papers of varying quality included in our systematic scoping review. For example, for the correlation between increased age and low PIF, papers supporting an association [11,17,21,22,25,28,30,32,34,35,44,47] were generally of higher quality (five of high quality, five of moderate quality and two of low quality; Supplementary Table S2) than the papers that did not support an association [18,19,29,36,46] (four of moderate quality and one of very low quality). Similarly, for gender, there were four papers of high quality and six of moderate quality supporting an association [16,17,18,19,22,26,32,35,36,47], but one of high quality, one of moderate quality, one of low quality and one of very low quality not supporting an association [11,21,29,34].

Four of four studies found a correlation between muscle strength parameters (handgrip and inspiratory muscle strength) with lower PIF, suggesting that handgrip strength could serve as a surrogate marker to predict PIF in clinical settings. Specific PIF meters are often not available in routine clinical care settings; as such, handgrip strength assessments, which are easy to administer and repeat, could therefore comprise one element in a pragmatic approach to assessing suitability for DPI use.

**Figure 2:**
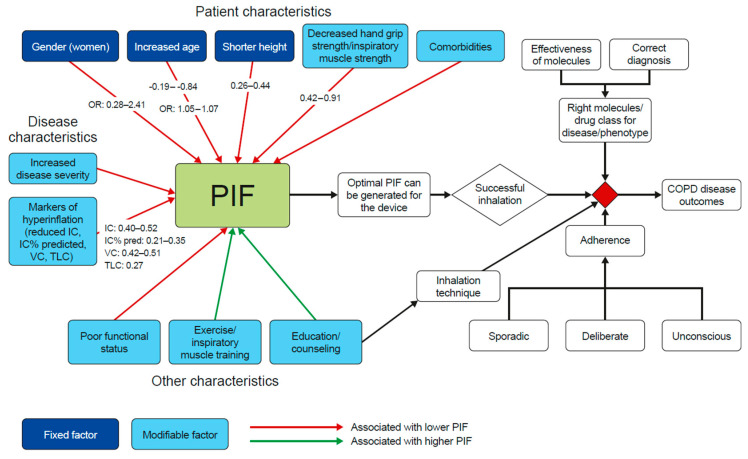
Impact of patient and disease factors on patient outcomes. Unless otherwise stated, data reported are R values; data only available for certain characteristics. Refer to Table 1 and the supplement for a full breakdown of the number of studies reporting correlation or risk estimates for each characteristic, as well as further detail on the nature of the statistical outputs calculated in each trial.

To put our findings into perspective, it is important to recognize that many of the patient factors and disease characteristics that we found to correlate with PIF are not modifiable (e.g., age and height) (Figure 2). Handgrip strength/mouth pressure, for example, may be modifiable using muscle training strategies, such as inspiratory muscle training, which is associated with improved PIF [26]. Choice of inhaler is also important; for example, metered-dose inhalers require good handgrip and coordination of inhalation with actuation of the device, which may be difficult in patients with reduced hand strength or impaired cognitive function [54,55]. There is evidence that finger strength differs considerably with age and comorbidities, which will impact the selection of the most appropriate inhaler [56]. The factors for healthcare professionals to consider to ensure effective drug delivery in elderly patients with COPD include both device factors, such as device type and complexity of use, and patient factors, such as inspiratory capabilities, manual dexterity, and hand strength, cognitive ability and comorbidities [55].

It is also important to consider the relationship between PIF, inhalation technique and adherence. Although the present systematic scoping review highlighted a scarcity of studies directly examining these relationships, inhalation technique and adherence are important factors that should be considered in all studies if we are to truly determine the relationship between PIF and disease outcomes.

Our systematic scoping review has some strengths and limitations. We believe this to be the first scoping literature review that has been carried out to identify the key determinants of PIF (patient factors and disease characteristics) in patients with COPD. In clinical settings where it is not possible to measure PIF against the simulated resistance of a particular device, the factors identified here may be valuable as a proxy to predict suboptimal PIF and determine the appropriateness of a prescribed inhaler. For example, an elderly woman with short stature and multiple comorbidities has a higher likelihood of having low PIF compared with a younger, taller male patient and may benefit from a flow-independent inhaler. However, as noted above, limitations include the heterogeneous nature of the data in terms of quality assessment, measurement of PIF with different devices, and differences in the classification of PIF as a binary or continuous variable, which impact the consistency of the findings. This is also why a meta-analysis with predictive scores could not be performed and findings were presented in a narrative manner. Another potential limitation is the assumption that the PIF values in each study are, in fact, a true reflection of patients’ maximum possible inspiratory flow (limited only by intrinsic physiologic factors such as airway tone and caliber). Although all studies were conducted under supervision in a clinic/laboratory setting, i.e., optimal conditions to allow patients to generate maximum inspiratory effort, it is possible that errors of inhalation technique (e.g., posture, angle of head) and/or psychologic factors (e.g., attention, motivation) may have influenced the readings. In terms of future research, further well-designed studies are needed to explore and quantitatively compare the strengths of the various predictors of PIF.

## 5. Conclusions

A review of available research exploring key determinants of PIF in patients with COPD, in terms of both patient factors and disease characteristics, has highlighted the complexity of the relationship between inhaler use and disease status. Low PIF has been shown to be associated with patient factors (increased age, female gender, shorter height, and decreased handgrip strength/inspiratory muscle strength) and disease characteristics (impaired lung function and increased disease severity). Even though further high-quality prospective studies that adequately adjust for potential confounders are needed, the characteristics identified in this review should be helpful for clinicians to identify the most appropriate inhaler type for their patients for those frequent situations when measurement of PIF against the simulated resistance of a DPI is not feasible. Studies such as the PIFotal study [57], which investigated the impact of PIF along with inhalation technique and medication adherence on health status in patients with COPD using a DPI, should provide additional insight on this important topic.

## Figures and Tables

**Table 1:** Patient and disease characteristics associated with PIF.

	Papers Evaluating Characteristic, *n*/Total Papers included in Review	Papers Supporting an Association between Characteristic and PIF, *n*/Total for Characteristic (% of Total for Characteristic)	Nature of Association Where Correlation is Observed	Papers not Supporting an Association Between Characteristic and PIF, *n*/Total for Characteristic (% of Total for Characteristic)
**Patient Characteristic**				
Age	17/35	12/17 (71%) [11,17,21,22,25,28,30,32,34,35,44,47] 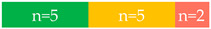	Age ↑; PIF ↓ ^1^ (r ranging from −0.19 to −0.84; odds ratio ranging from 1.05 to 1.07)	5/17 (29%) [18,19,29,36,46] 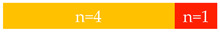
Gender	14/35	10/14 (71%) [16,17,18,19,22,26,32,35,36,47] 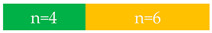	Female gender; PIF ↓ ^2^ (odds ratio ranging from 0.28 to 2.41)	4/14 (29%) [11,21,29,34] 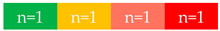
Height	11/35	8/11 (73%) [16,17,18,19,22,28,32,44] 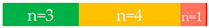	Height ↓; PIF ↓ ^3^ (r ranging from 0.26 to 0.44)	3/11 (27%) [11,25,35] 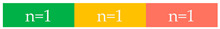
Body weight/body mass index/body composition	12/35	2/12 (17%) [17,37] 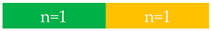	Body weight ↓; PIF ↓ [17] ^4^ (r 0.22) Fat-free mass ↓ [37]; PIF ↓ (r_pb_ −0.58)	10/12 (83%) [11,18,19,22,28,29,32,34,36,44] 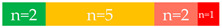
Handgrip strength/inspiratory muscle strength	4/35	4/4 (100%) [21,26,27,28] 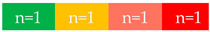	Handgrip strength ↓; PIF ↓ [21] (effect size in adjusted model: 0.49 [95% CI 0.08, 0.90]) Maximal inspiratory mouth pressure (a measure of inspiratory muscle strength) ↓; PIF ↓ [26,27,28] ^5^ (r ranging from 0.42 to 0.91)	0/4 (0%)
Comorbidities	4/35	3/4 (75%) [21,32,35] 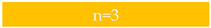 Note: While these papers each include a correlation between PIF and at least one comorbidity, they also contain data showing no correlation between PIF and other comorbidities	Asthma [21]; anemia and coronary artery disease [32]; pneumonia and ischemic heart disease [35]; PIF ↓	1/4 (25%) [11] 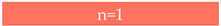
Smoking status	5/35	1/5 (20%) [35] 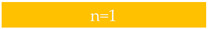	Current smoker; PIF ↓ (Odds ratio: 2.06)	4/5 (80%) [21,29,34,36] 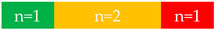
Ethnicity	3/35	0/3 (0%)	N/A	3/3 (100%) [11,21,32] 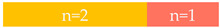
**Disease characteristics**				
IC	2/35	2/2 (100%) [16,27] 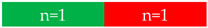	IC ↓; PIF ↓ (r ranging from 0.40 to 0.52)	0/2 (0%)
IC% pred.	4/35	3/4 (75%) [11,16,22] 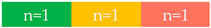	IC% pred ↓; PIF ↓ (r ranging from 0.21 to 0.35)	1/4 (25%) [27] 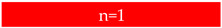
FEV_1_	14/35	9/14 (64%) [16,17,19,25,32,34,36,44,48] ^6^^ 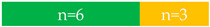 ^	FEV_1_ ↓; PIF ↓ (r ranging from 0.29 to 0.73)	5/14 (35%) [18,28,29,35,41] 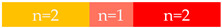
FEV_1_% pred.	16/35	3/16 (19%) [30,32,44] 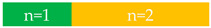	FEV_1_ % pred ↓; PIF ↓ (r ranging from 0.22 to 0.68)	13/16 (81%) [11,16,17,19,22,27,28,29,34,35,36,41,43] 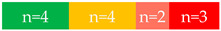
FVC	10/35	7/10 (70%) [16,25,28,32,34,36,44] ^6,7^^ 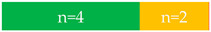 ^	FVC ↓; PIF ↓ [16,25,28,32,36,44] ^6,7^ (r ranging from 0.29 to 0.48; odds ratio: 0.961) [34]	3/10 (30%) [18,19,35] 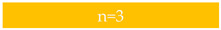
FVC% pred.	11/35	3/11 (27%) [16,22,34] 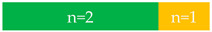	% pred FVC ↓; PIF ↓ (r 0.37)	8/11 (73%) [11,19,27,28,29,35,36,44] 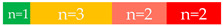
PEF	5/35	5/5 (100%) [17,19,28,44,48] 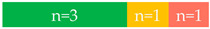	PEF ↓; PIF ↓ (r ranging from 0.32 to 0.80)	0/5 (0%)
PEF% pred.	2/35	0/2 (0%)	N/A	2/2 (100%) [17,27] 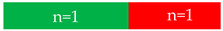
TLC	2/35	2/2 (100%) [16,48] 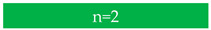	TLC ↓; PIF ↓ (r 0.27)	0/2 (0%)
TLC% pred.	2/35	0/2 (0%)	N/A	2/2 (100%) [16,27] 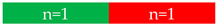
DL_CO_	1/35	1/1 (100%) [48] 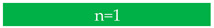	DL_CO_ ↓; PIF ↓ (r 0.79)	0/1 (0%)
VC	1/35	1/1 (100%) [27] 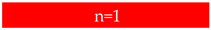	VC ↓; PIF ↓ (r ranging from 0.42 to 0.51)	0/1 (0%)
VC% pred.	1/35	0/1 (0%)	N/A	1/1 (100%) [27] 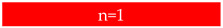
MEP	2/35	1/2 (50%) [28] ^ 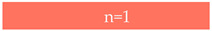 ^	MEP ↓; PIF ↓ (r 0.5)	1/2 (50%) [27] 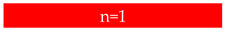
FIV	1/35	1/1 (100%) [19] ^ 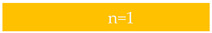 ^	FIV ↓; PIF ↓ (r not stated)	0/1 (0%)
FIVC	2/35	2/2 (100%) [19,44] ^ 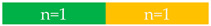 ^	FIVC ↓; PIF ↓ (r 0.63)	0/2 (0%)
MVV	1/35	1/1 (100%) [19] ^ 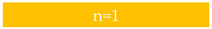 ^	MVV ↓; PIF ↓ (r not stated)	0/1 (0%)
FIF_max_	1/35	1/1 (100%) [16] 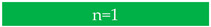	FIF_max_ ↓; PIF ↓ (r 0.65)	0/1 (0%)
RV% pred.	2/35	0/2 (0%)	N/A	2/2 (100%) [16,27] 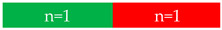
RV/TLC ratio	1/35	1/1 (100%) [16] 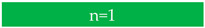	RV/TLC ratio ↑; PIF ↓ (r −0.19)	0/1 (0%)
Disease severity	11/35	9/11 (82%) [19,26,30,33,40,41,47,48,50] ^8^ 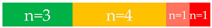	Disease severity ↑; PIF ↓	2/11 (18%) [18,36]^ 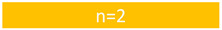 ^
Exacerbations	3/35	1/3 (33%) [34] 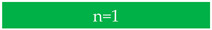	≥2 exacerbations in previous year; PIF ↓	2/3 (67%) [32,45] 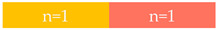
Symptoms (CAT, mMRC)	7/35	2/7 (29%) [11,19] 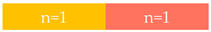	CAT scores ↑; PIF ↓ [11,19]	5/7 (71%) [18,21,32,34,35] 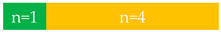
**Other factors**				
Enhanced educational training/instruction/verbal training/counseling	5/35	5/5 (100%) [31,33,49,50,51] ^9^ 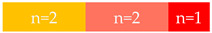	Enhanced educational training/instruction/verbal training/counseling ↑; PIF ↑	0/5 (0%)
Exercise training/inspiratory muscle training/respiratory physiotherapy	3/35	2/3 (67%) [26,52] 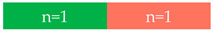	Exercise training ↑; PIF ↑ [52] Inspiratory muscle training ↑; PIF ↑ [26]	1/3 (33%) [39] 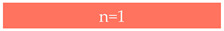
Critical errors in inhalation technique/non-adherence	1/35	1/1 (100%) [34] 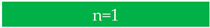	Inhalation technique errors ↑; PIF ↓ Unconscious non-adherence ↑; PIF ↓ Deliberate non-adherence ↓; PIF ↓ Erratic non-adherence ↓; PIF ↓	0/1 ^10^ (0%)
ADL score	1/35	1/1 (100%) [32] ^ 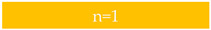 ^	ADL score ↑; PIF ↓	0/1 (0%)
Length of stay	1/35	0/1 (0%)	N/A	1/1 (100%) [11] 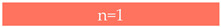
ICS use	1/35	0/1 (0%)	N/A	1/1 (100%) [11] 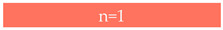
Primary education	1/35	0/1 (0%)	N/A	1/1 (100%) [34] 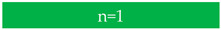
FSI-10	1/35	0/1 (0%)	N/A	1/1 (100%) [34] 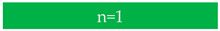
Pursed lip breathing	1/35	0/1 (0%)	N/A	1/1 (100%) [38] 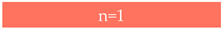
LVEF	1/35	0/1 (0%)	N/A	1/1 (100%) [11] 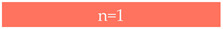
Airway conductance	1/35	0/1 (0%)	N/A	1/1 (100%) [42] 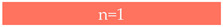
Arterial pH, PO_2_/FiO_2_	1/35	0/1 (0%)	N/A	1/1 (100%) [29] 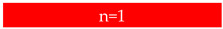

For papers reporting an association, R values and odds ratio are quoted where they are stated in the paper. ^1^ Altman et al. [47] noted a correlation between higher age and reduced PIF but no statistical analysis was reported. ^2^ Altman et al. [47] noted a correlation between female gender and reduced PIF but no statistical analysis was reported; Duarte et al. [16] noted a correlation between gender and PIF but did not state which gender was associated with reduced PIF and no statistical analysis was reported. ^3^ Duarte et al. [16] noted a correlation between reduced height and reduced PIF, but no statistical analysis was reported; for Ghosh et al. [18], the correlation was only significant at low-medium resistance. ^4^ Malmberg et al. [17] noted a significant correlation between weight and PIF, but the weight had insignificant effects when added to a linear regression model for best prediction of PIF values. ^5^ Terzano et al. [27] noted a mild correlation between maximal inspiratory pressure and PIF but no statistical analysis was reported; for Janssens et al. [28], the correlation was noted in the total study group (COPD and control patients). ^6^ Farkas et al. [36] noted a correlation between FEV_1_ and FVC for Breezhaler and Turbuhaler, but not for Genuair. ^7^ Note that for Duarte et al. [16], FVC was higher in patients with lower PIF. ^8^ Altman et al. [47], Weiner et al. [26], Chodosh et al. [41], Broeders et al. [50], Magnussen et al. [40], and Al-Showair et al. [33] noted a correlation between increasing disease severity and reduced PIF, but no statistical analysis was reported. ^9^ Nsour et al. [51] noted a correlation between counseling and increased PIF but no statistical analysis was reported. ^10^ Represas-Represas et al. [34] also reported that the association between PIF and intermediate or bad adherence was non-significant. ADL, activities of daily living; CAT, COPD Assessment Test™; CI, confidence interval; DL_CO_, diffusing capacity of the lung for carbon monoxide; FEV_1_, forced expiratory volume in 1 second; FIF_max_, maximal forced inspiratory flow; FiO_2_, a fraction of inhaled oxygen; FIV, forced inspiratory volume; FIVC, forced inspiratory vital capacity; FSI-10, Feeling of Satisfaction with Inhaler questionnaire; FVC, forced vital capacity; IC, inspiratory capacity; ICS, inhaled corticosteroids; N/A, not applicable; LVEF, left ventricular ejection fraction; MEP, maximal expiratory pressure; mMRC, modified British Medical Research Council dyspnea scale; MVV, maximum voluntary ventilation; PEF, peak expiratory flow; PIF, peak inspiratory flow; PO_2_, partial pressure of oxygen; pred., predicted; RV, residual volume; TLC, total lung capacity; VC, vital capacity.

## Supplementary Materials

The following are available online at https://www.mdpi.com/article/10.3390/biomedicines10020458/s1, Supplementary Methods, Table S1: Search terms, Table S2: Definitions of quality, Table S3: Quality assessment and Table S4: Data charting.
